# Histology and morphometry of the skin of purple spaghetti-eel *Moringua raitaborua* (Anguilliformes, Moringuidae)

**DOI:** 10.1186/s42649-023-00093-6

**Published:** 2023-10-31

**Authors:** Hyun-Tae Kim

**Affiliations:** Department of Science Education, Jeonju National University of Education, Jeonju, 55101 Republic of Korea

**Keywords:** Skin, Histology, Morphology, Morphometry, *Moringua raitaborua*, Cutaneous respiration

## Abstract

The purple spaghetti-eel *Moringua raitaborua* lives on the sandy or muddy bottoms of estuaries, which are subject to rapid and wide changes in salinity, pH, and osmoregulatory and hypoxic conditions due to the influx of organic materials from sources of freshwater. The species has adapted to hypoxic environments by developing a thicker epidermis with stratified polygonal cells, club cells, two types of mucous cells (goblet and, oval cells), stratified cuboidal cells and dermis with abundant blood capillaries. Among them, histological modification of thinner dorsal, lateral, and ventral body skin to include abundant capillaries and well-developed dermal vascularization may provide cutaneous respiration, permitting survival in brackish waters with low levels of oxygen and variable environmental parameters.

## Introduction

Teleost species of fish exchange dissolved oxygen and carbon dioxide in aquatic environments through diverse respiratory adaptations such as gills (Park et al. 2014), skin (Glover et al. 2013), intestines (Park and Kim 2001), labyrinth organs (Zaccone et al. 2019), buccal cavities (Zaccone et al. 2018), swim bladders or hydrostatic organs (Zaccone et al. 2012), opercula (Summerfelt and Smith 1990), and lungs (Glass and Rantin 2009). Among them, the skin is a significant respiratory mediator that enables teleosts to absorb 5 to 30% of supplementary oxygens (Nilsson et al. 2004; Kim and Park 2011). This percentage rises to 50% in amphibious mudskippers, which spend much of their lives in air (Graham 2011). Teleosts have histologically adapted skin to allow for cutaneous respiration through the following mechanisms (Beon et al. 2013; Glover et al. 2013; Kim 2022): a thicker epidermis with diverse gland and large cells; the presence of intraepithelial blood capillaries; a defined lymphatic space at the basal layer of epidermis; well-vascularized connective tissue, an absence of scales; and other specific multicellular adaptations related to gas exchange.

The purple spaghetti eel *Moringua raitaborua*, which has an elongated and stubby body migrates from freshwater to seawater habitats in tropical zones in Nepal, India, Bangaladesh, and Philippines as they mature, and is most often found burrowed in the muddy bottoms of estuaries (Menes et al. 2010; Kottelat 2013; Behera et al. 2021). In general, coasts and estuarine zones have hypoxic water (2.8 mg O^2^ L^-1^ or lower) caused by excessive nutrient runoff, algal blooms, and stagnant water during dry season (Zhang et al. 2013; Mishra 2020). With such environmental conditions, brackish water–dwelling fishes are exposed to considerable skin stress that requires physiological tolerance of rapid changes in salinity, dissolved oxygen levels, pH, and water volume (Hu and Cai 2013; Robbins and Lisle 2018). While researching the histology of fishes inhabiting intertidal pools and estuarine standing water, dermal vascularization (as in *Rhinogobius brunneus* and *Tridentiger brevispinis*) was found in the skin of *M*. *raitaborua* (Kim 2022; Kim et al. 2022). This study aims to describe the skin structure and analyze the morphometry of the epidermal thickness and diffusion distance of *M*. *raitaborua*, along with their relevance to cutaneous respiration.

## Materials and methods

### Specimen collection

Five adult *M*. *raitaborua* individuals (20.2, 24.6, 28.3, 28.5, 32.0 cm in standard length, respectively) were purchased at a fish market (Aquapro) after being imported from India on December 16, 2021. For examination by light microscopy, the specimens were fixed in a 10% neutral buffered formalin solution at pH 7.4 for 1 day after receiving 0.05% tricaine methanesulfonate (MS-222, Sigma, St. Louis, MO, USA) as anesthesia in the laboratory. The experimental procedures strictly followed the rules of Jeonbuk National University Institutional Animal Care and Use Committee for animal ethics (License Number: CBNU-2023-00060).

### Microscopic investigation

**Fig. 1:**
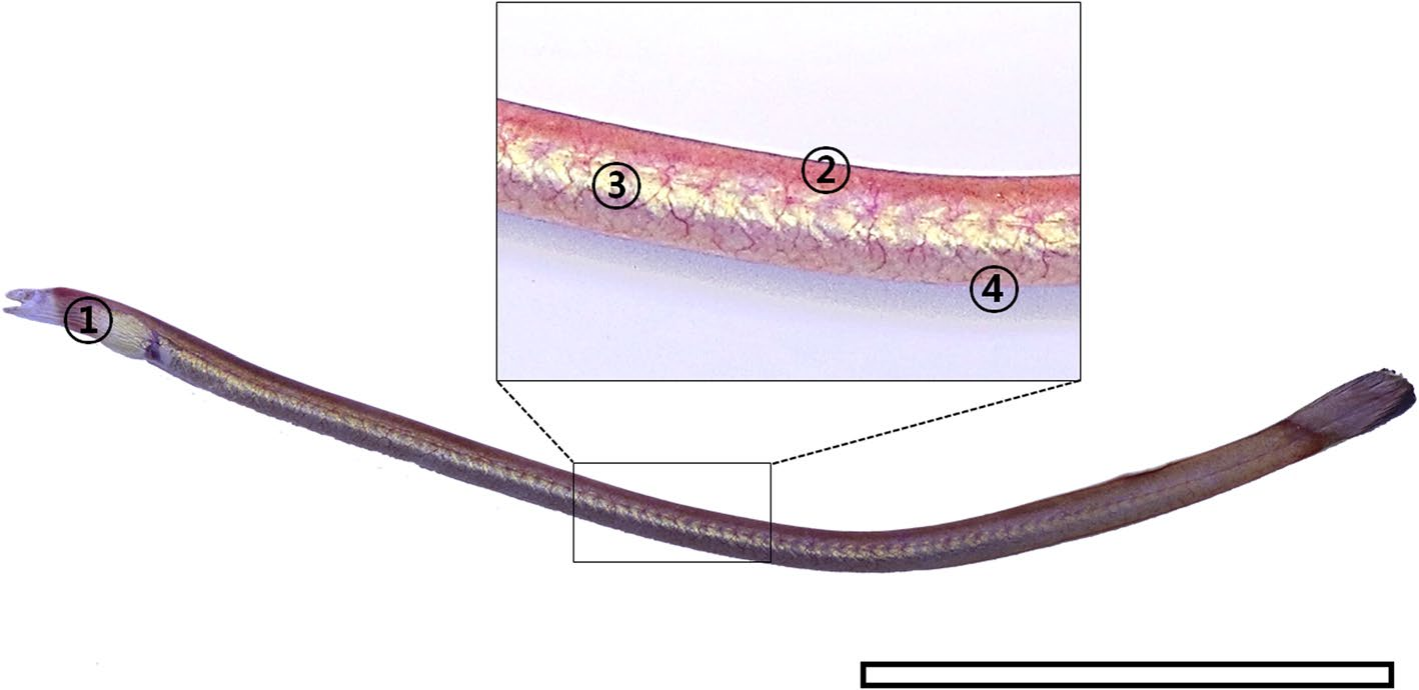
The photograph of *Moringua raitaborua*. Each number indicates sectioned regions of the skin. The bar indicates 10 cm. ①, operculum; ②, dorsal body; ③, lateral body; ④, ventral body

Each skin region (operculum, dorsal body, lateral body, ventral body; Fig. 1) of *M*. *raitaborua* specimens fixed with formalin solution was dissected to approximately 0.5 cm^2^, respectively. Each tissue was processed through an ascending series of concentrations (50–100%) of alcohol for 1 h, cleared with xylene, and then embedded in ordinary paraffin at 65 ℃. The paraffin-embedded tissue blocks were serially sectioned at 5 cm intervals with a microtome (Jung Histocut, model 820-II, Leica, Wetzlar, Germany) and mounted on microscope glass slides. Each section was then deparaffinized in xylene, dehydrated through descending alcohol concentrations (100–50%), and stained with hematoxylin and eosin (H&E) and Masson’s trichrome to confirm blood capillaries, connective tissue, basement membranes, and specific cells. Images of stained tissues were acquired with a light microscope (Imager A1, Carl Zeiss, Germany) and analyzed in Axio Vision (LE REL. 4.5, Carl Zeiss).

### Statistical analysis

A regional comparison of epithelial thickness and diffusion distance (the shortest distance from a capillary to the skin’s surface) of each skin sample was performed using PASW SPSS statistical software (SPSS version 18.0, IBM, Armonk, NY, USA). The normality and homogeneity of variance for all samples was verified by Kolmogorov–Smirnov or Shapiro–Wilk test and Levene’s test (*P* > 0.05). One-way analysis of variance (ANOVA) with Tukey’s honestly significant difference test was used to compare the mean of data for epithelial thickness and diffusion distance. An analysis of covariance (ANCOVA) was utilized for statistical analysis of diffusion distance based on covariate epithelial thickness. The Pearson correlation coefficient was used to determine the positive linear association between two factors.

## Results

### Histology

**Fig. 2:**
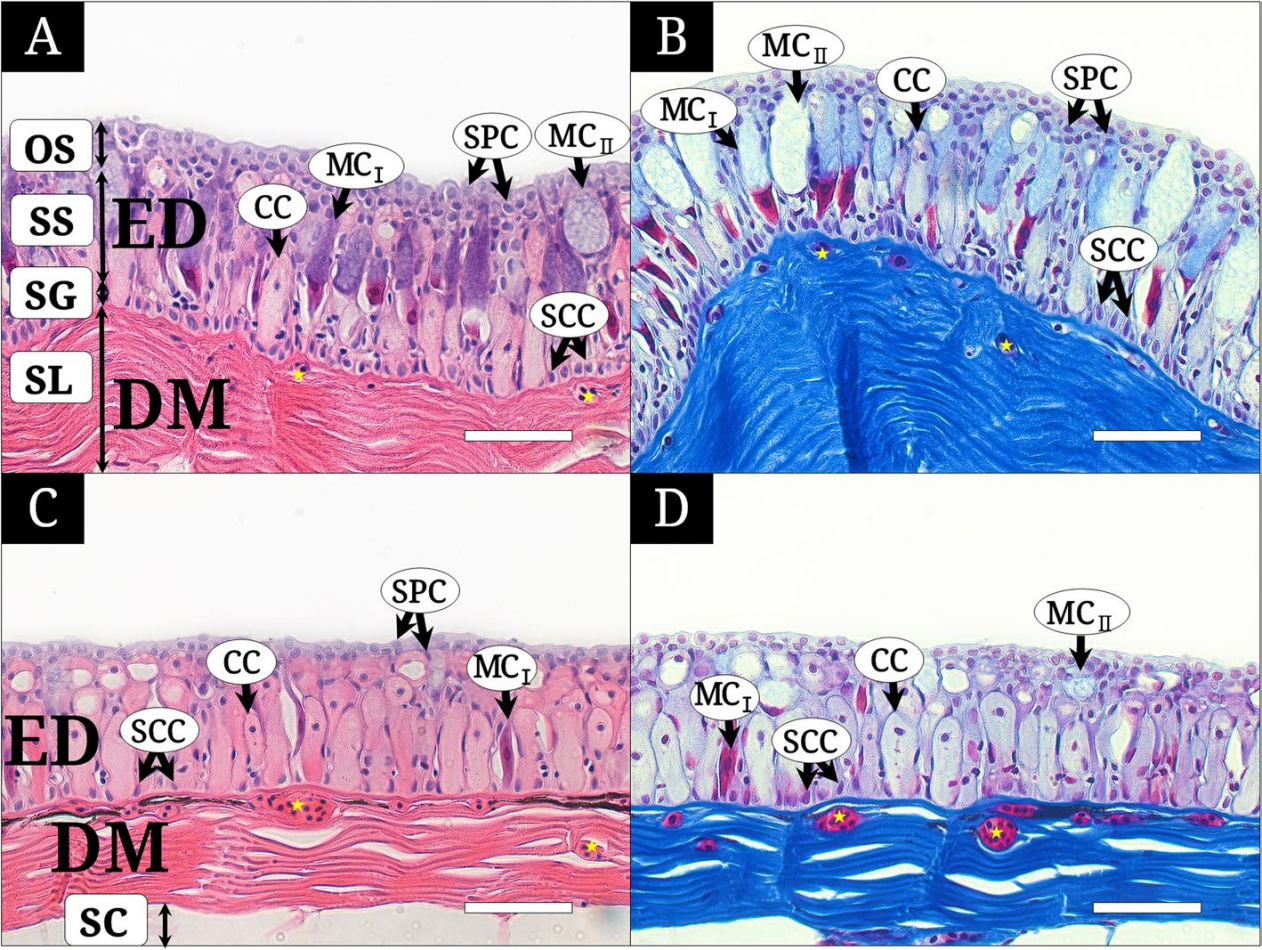
Histological characteristics of the operculum and dorsal body skin of *Moringua raitaborua*, stained with Hematoxylin and Eosin (**A** and **C**), Masson’s trichrome (**B** and **D**). **A** and **B**, the operculum consisting of the epidermis (ED) having the outermost surface layer with stratified polygonal cells (SPC), the stratum spinosum with club cells (CC), two types of mucous cells (MC_I_ and MC_II_), and the stratum germinativum (SG) with stratified cuboidal cells (SCC), the dermis (DM) having the stratum laxum (SL) with blood capillaries (yellow asterisk) and the stratum compactum (SC); C and D, the dorsal body having ED with SPCs, CCs, MC_I_, MC_II_, SCCs and DM having SL with abundant blood capillaries. All bars indicate 50 μm

**Fig. 3:**
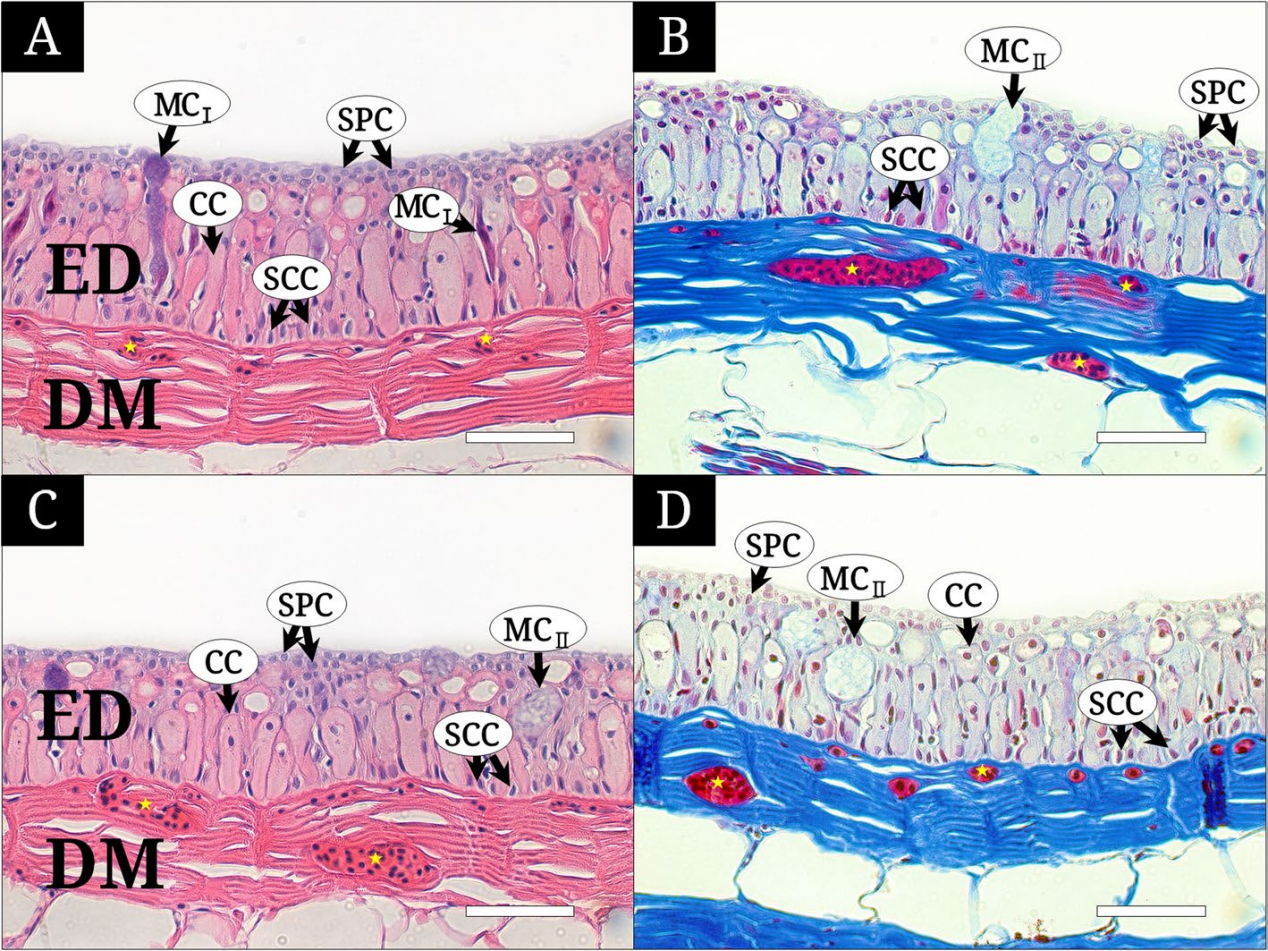
Histological characteristics of the lateral and ventral body skin of *Moringua raitaborua*, stained with Hematoxylin and Eosin (**A** and **C**), Masson’s trichrome (**B** and **D**). **A** and **B**, the lateral body consisting of the epidermis (ED) with stratified polygonal cells (SPC), club cells (CC), two types of mucous cells (MC_I_ and MC_II_), stratified cuboidal cells (SCC) and the dermis (DM) with blood capillaries (yellow asterisk); **C** and **D**, the ventral body consisting of ED with SPC, CC, MC_II_, SCC and DM with blood capillaries. All bars indicate 50 μm

The skin of *M*. *raitaborua* individuals was classified into two main parts, the epidermis (ED) and dermis (DM), which are separated by a basement membrane (Figs. 2 and 3).

The epidermis consisted of the outermost surface layer (OS), stratum spinosum (SS), and stratum germinativum (SG) (Fig. 2A). The OS is an upper region built of stratified polygonal cells (SPC). The SS is a thicker region with diverse multi-cells such as club cells (CCs), and mucous cells (MCs). The SG consisted of a single basal layer of stratified cuboidal cells (SCCs) at the bottom of the ED. The SPCs had a polygonal cell with a violet nucleus and faint cytoplasm when stained with H&E, and a purple nucleus and weak blush cytoplasm when stained with Masson’s trichrome (Figs. 2A–C and 3). The CCs had a wide cylindrical body with small violet nucleus and pink cytoplasm by H&E and small violet nucleus and faint cytoplasm by Masson’s trichrome, and constituted most of the SS in the dorsal body, lateral body, and ventral body (Figs. 2 and 3). The MCs were classified into two types of unicellular gland cell (MC_I_ and MC_II_). MC_I_ was an elongated tubular cell extending from the basement membrane to the surface and showed an upper part (purple with H&E and blush with Masson’s trichrome) and a reddish lower part of the cell body and the basal position of nucleus. These were more abundant and larger in the operculum than in other skin regions (Fig. 2 A). MC_II_ had a flattened nucleus at the cell bottom and wide cytoplasm (hazy color with H&E and blush with Masson’s trichrome) (Figs. 2A, B, and D and 3B, C, and D). The SCCs were small but densely arranged cells on the basement membrane, with a larger nucleus and narrower cytoplasm (Fig. 2).

The DM comprised the SL and SC. The dermis contained a few capillaries, with one to four blood cells just below the basement membrane in the operculum (Fig. 2A and B), and featured well-developed vascularization, with numerous blood cells among dermal collagen fibers the dorsal, lateral, and ventral body (Figs. 2C–D and 3).

### Morphometry

**Fig. 4:**
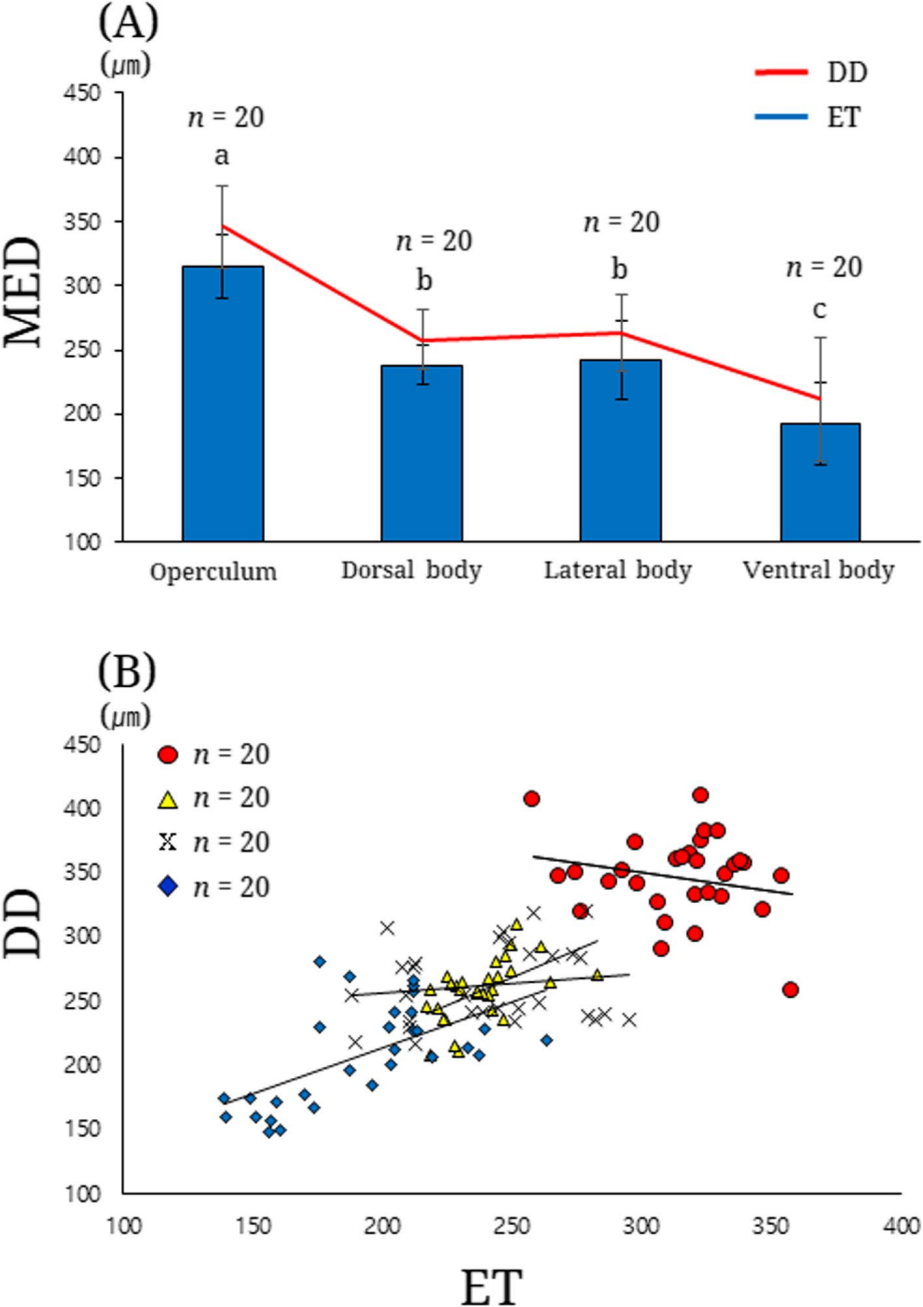
Regional comparison of epithelial thickness and diffusion distance of *Moringua raitaborua* skin. **A** line and bar graphs for relative difference between four skin regions (operculum, dorsal body, lateral body, ventral body); **B** a scatterplot graph showing a correlation between epithelial thickness (x-axis, *n* = 20) and diffusion distance (y-axis, *n* = 20) in each skin region. Red circle, operculum; yellow triangle, dorsal body; X, lateral body; blue diamond, ventral body. DD, diffusion distance; MED, a measured value of epithelial thickness and diffusion distance

Measurement of epithelial thickness revealed a regional difference: the operculum was the thickest (mean = 315.4 ± standard deviation [SD] = 24.7; range = 258.5–358.0), with the lateral body (241.9 ± 30.6; 187.9–295.2) and dorsal body (238.0 ± 15.1; 216.8–283.0) exhibiting similar values, and the ventral body was the thinnest (191.9 ± 32.1; 139.1–263.1). These measurements showed a highly significant difference in epithelial thickness (one-way ANOVA, *df* = 3, *f* = 111.457, *p* < 0.001; Fig. 4A). The diffusion distance also included a relative difference between four regions: the operculum was associated with the highest value (346.1 ± 32.0; 257.3–409.2), followed by the lateral body (262.9 ± 30.3, 216.5–319.7), dorsal body (258.1 ± 23.5; 208.7–309.5), ventral body (208.1 ± 38.7, 148.8–281.5). These showed a highly significant difference in diffusion distance (one-way ANOVA, *d f* = 3, *f* = 98.259, *p* < 0.001; Fig. 4A). The diffusion distance between the four skin regions was strongly affected by epithelial thickness as a covariate (ANCOVA, *df* = 3, *f* = 13.671, *p* < 0.001; Fig. 4B). The two factors were highly and positively correlated in the four skin regions (Pearson’s correlation coefficient, *r* = 803, *p* < 0.001; Fig. 4B).

## Discussion

Fish skin is a multi-functional envelope that acts as physical barrier to potential bacterial infections (Zhang et al. 2021), abrasion (Lv et al. 2023), sensory system (Mogdans 2019), color expression (Vissio et al. 2021), much more freedom motion (Clark et al. 2016), acid-base regulation (Perry and Gilmour 2006), excretion of nitrogenous compounds (Wood 1993), and osmoregulation (Marshall 2012). Such physiologies are well-supported by the ED with SPCs, CCs, MCs, and SCCs confirmed in this study. Among them, two unicellular secretary glands, CCs and MCs, not only produce alarm-clue chemicals (proteins and pheromones such as serotonin and 5-HT) with cytoplasmic membrane breakage for antipredator response in conspecifics (Zaccone et al. 1990; Carreau-Green et al. 2008; Manek et al. 2013) but also engage in defense against pathogens that can penetrate the skin (Pollock 2011) and repair damaged tissues with chondroitin and keratin (Damasceno et al. 2012). They can help oxygen penetrate deeper toward the dermal matrix of connective tissue due an abundance of water and acidophilic proteins of a positive ion (Jakubowski 1958; Mittal and Munshi 1971; Park 2002). These reports indicate that CCs and MCs of *M*. *raitaborua* may constitute a cytological delivery system for efficient oxygen diffusion or storage in cutaneous respiration, and act as a skin protector against harmful substances a fish migrating can expect in encounter in contaminated habitats.

*M*. *raitaborua* also had two types of MCs: elongated (MC_I_, ii) and oval (MC_II_). The elongated MC_I_ is a goblet mucous cell due to its nucleus position, cell morphology, and histochemistry, and has been reported in the skin of other teleosts (Rakers et al. 2011; Mohamed et al. 2020; Abolfathi et al. 2022). Fishelson (1996) noted that abundant goblet cells of the skin of the marine eel *Siderea grisea* skin are relevant to skin-damage reduction during movement on a hard substrate and the initiation of digging into the substrate. Elsheikh (2012) confirmed that goblet cell secretion of *Oreochromis niloticus* protects the epidermis of the buccal cavity from physical abrasion during feeding. These findings support the presence of more MC_I_s of the operculum of *M*. raitaborua, which feeds on burrowing fish or invertebrates living in the sand and dig into bottom substrate using its head as ecology, at least in genus *Moringua* (Smith 1997).

Many amphibious fishes exhibiting cutaneous respiration contain a thicker ED produced by large secretary cells as follows: 38.4–156.8 μm thick, a freshwater goby *Rhinogobius brunneus* (Kim et al. 2022); 35.4–150 μm, a trident goby *Tridentiger brevispinis* (Kim 2022); 136.3–195.5 μm, a mud loach *Misgurnus mizolepis* (Park et al. 2001); 146–495 μm, a torrent catfish *Liobagrus mediadiposalis* (Park et al. 2003a); 59.0 μm 297.0 μm, an eel goby *Odontamblyopus lacepedii* (Park et al. 2003b), M. *raitaborua* (246.8 ± 51.5 μm, 139.1–358.0; mean ± SD, range) with CCs and MCs. Reduced diffusion distance of the skin also is strong evidence that confirms more rapid gas-exchange, as measured by an ascending vascularization that represents two histological categories in its occurrence position (Glover et al. 2013): (i) intraepidermal blood capillaries of the outermost surface layer (*Mastcembellus pancalus* with a mean diffusion distance of 34.0 μm; Mittal and Munshi 1971; *Periophthalmus modestus* with a mean of 1.4 μm; Park et al. 2000), the middle layer (*Liobagrus mediadiposalis* with a mean of 169 μm; Park et al. 2003a), and the stratum germinativum (*Rhinogobius brunneus* with a range of 35.0–202.6 μm; Kim et al. 2022), and (ii) well-developed dermal vascularization among collagen fibers of SL just below the basement membrane (*Pseudobagrus brevicorpus*, with a range of 19.9–399.4 μm, Park et al. 2010; abd *Tridentiger brevispinis*, with a range of 51.4–216.9, Kim 2022) (Kazerouni and Khodabandeh 2010; Romano et al. 2019). In this study, *M*. *raitaborua* showed reduced diffusion distance (268.8 ± 58.7 μm), which was similar to and affected strongly by ET (covariance, *P* < 0.001) in all skin regions, indicating that capillaries of *M*. *raitaborua* can get close to the basement membrane of the SL. For such a histological character, Park et al. (2003a) suggest that a reduced diffusion distance (mean = 169 μm, range = 22.5–220) by dermal vascularization as well as intraepidermal blood capillaries in *L*. *mediadiposalis* are meaningful histological modifications for fish that enable them to survive in frequently hypoxic habitats. Ba-Omar and AI-Riyami (2009) reported that rich dermal vascularization below the epidermis and in the dermis of an amphibious benny, *Istiblennius edentulous*, facilitates efficient gas exchange for cutaneous respiration. Thinner dorsal, lateral, and ventral bodies of *M*. *raitaborua* with reduced diffusion distance by ascending blood capillaries and well-developed vascularization may be collectively represent major skin region for gas-exchange and the supply of deficient oxygen through cutaneous respiration.

## Conclusions

The purple spaghetti eel *M. raitaborua* has a thicker epidermis (the operculum was the thickest, at 315.4 ± 24.7, 258.5–358.0 [mean ± SD, range], the ventral body was the thinnest at 191.9 ± 32.1, 139.1–263.1) with stratified polygonal cells, club cells, and two types of mucous cells: elongated MC_I_ goblet cells feature an upper part (purple with H&E staining and blush with Masson’s trichrome staining) and a reddish lower part of the cell body, and the basal position of nucleus, whereas MC_II_ oval cells include a flattened nucleus at the cell bottom and wide cytoplasm (hazy color with H&E staining and blush with Masson’s trichrome staining), and stratified cuboidal cells. In particular, the dermis just below basement membrane in dorsal body, lateral body, and ventral body regions have abundant blood capillaries and well-developed dermal vascularization. These findings demonstrate the eel’s adaptation to cutaneous respiration to obtain supplementary oxygen in hypoxic muddy regions of brackish-water estuaries.

## Data Availability

Not applicable.

## Abbreviations

CC: Club cell
DM: Dermis
ED: Epidermis
MC: Mucous cell
OS: Outermost surface layer
SC: Stratum compactum
SCC: Stratified cuboidal cell
SG: Stratum germinativum
SL: Stratified laxum
SPC: Stratified polygonal cell
SS: Stratum spinosum
